# Tobacco chewing and female oral cavity cancer risk in Karunagappally cohort, India

**DOI:** 10.1038/sj.bjc.6604907

**Published:** 2009-03-03

**Authors:** P A Jayalekshmi, P Gangadharan, S Akiba, R R K Nair, M Tsuji, B Rajan

**Affiliations:** 1 Regional Cancer Center, Trivandrum, Kerala, India; 2 Natural Background Radiation Cancer Registry, Karunagappally, Kerala, India; 3 Amrita Institute of Medical Sciences, Cochin, Kerala, India; 4 Kagoshima University Graduate School of Medical and Dental Sciences, Kagoshima, Japan; 5 Kumamoto University Graduate School of Medical Sciences, Kumamoto, Japan

**Keywords:** tobacco chewing, oral cancer, India

## Abstract

This study examined oral cancer in a cohort of 78 140 women aged 30–84 years in Karunagappally, Kerala, India, on whom baseline information was collected on lifestyle, including tobacco chewing, and sociodemographic factors during the period 1990–1997. By the end of 2005, 92 oral cancer cases were identified by the Karunagappally Cancer Registry. Poisson regression analysis of grouped data, taking into account age and income, showed that oral cancer incidence was strongly related to daily frequency of tobacco chewing (*P*<0.001) and was increased 9.2-fold among women chewing tobacco 10 times or more a day. The risk increased with the duration of tobacco chewing during the first 20 years of tobacco chewing. Age at starting tobacco chewing was not significantly related to oral cancer risk. This is the first cohort study of oral cancer in relation to tobacco chewing among women.

Globally, oral cancer is the 11th most common cancer and is responsible for about 200 000 deaths each year (IARC, 2003), two-thirds of which were in economically developing countries. Tobacco chewing as a cause for oral cancer was suggested as early as the beginning of the last century (Niblock, 1902; Orr, 1933). To date, epidemiological studies conducted in South Asia, west Europe and North America have clearly shown the relationship between oral cancer risk and tobacco chewing among men (Critchley and Unal, 2003; IARC, 2007). However, to our knowledge, the corresponding risk in women has been examined only by a few studies.

In this study, we analysed the oral cancer risk among women in relation to tobacco use, and socioeconomic status (SES) in a rural cohort in Kerala. To our knowledge, this is the first cohort study to examine the association of oral cavity cancer risk with tobacco chewing among women. It is relevant that smoking and alcohol drinking were rare in this women population.

## SUBJECTS AND METHODS

In the early 1990s, a cohort was established of virtually all the residents in Karunagappally (Nair *et al*, 1999), a rural coastal area in Kollam district of Kerala, south west India. This taluk consisted of 12 panchayats at taluk being an administrative unit, corresponding to a county, with panchayats as subunits. According to the 1991 Census, this taluk had a population of 385 103 (191 149 males and 193 954 females) residing in an area of 192 km^2^ All the households (*N*=71 674) in Karunagappally taluk were visited by 12–14 trained interviewers, starting from 1 January 1990 and ending on 31 December 1997 (Jayalekshmi *et al*, 2008). Using a 6-page standardised questionnaire, they collected information on sociodemographic factors, religion, family income in rupees, education, occupation, lifestyles and other factors. Residents were asked if they never chewed tobacco, habitually chewed it in the past or habitually chewed it currently. For those who ever habitually chewed tobacco, further questions were asked on the daily frequency, age at starting and the duration. For ex-chewers, age at stopping was also asked. The same types of questions were asked to beedi and cigarette smokers.

In total, this household survey collected personal information on 359 614 subjects in 71 674 households, which correspond to 93% of population and 94% of households in Karunagappally by the 1991 census. There were 81 514 women aged 30–84 years old at the time of interview. We excluded the following from analysis: those younger than 30 years of age, as cancer risk is low in this age range; those aged 85 years or older; workers employed in the local Rare Earth factory, who might have various occupational exposures (*N*=29); 166 subjects who had died or been diagnosed as cancer before the base-line interview; and those who died within 3 years of interview, as their lifestyles might have been affected by their health conditions. Thus, there were 79 593 subjects for statistical analysis.

The entry into the cohort was 1 January 1990 or the date of interview, which was started on 1 January 1990 and ended on 31 December 1997. A cohort member was censored when she was (i) diagnosed as cancer other than oral cancer, (ii) died of causes other than oral cancer or (iii) migrated from the study area. Thus, the end of follow-up was the date of diagnosis for cancer cases, of death for those deceased, of the end of follow-up (31 December 2005), of moving out, or reaching the age of 85 years. In person-year calculation, we used the information on migration of cohort members even though this was available only for a part of our observation period; this caused only small changes in relative risk estimates.

In this study, we analysed cancer incidence in the period 1990–2005. Cancer cases among the cohort were ascertained by the cancer registry in Karunagappally, which was officially initiated as of 1 January 1990 and has been reported in ‘Cancer Incidence in Five Continents’, vols. VII–IX (Nair *et al*, 1997, 2002; Jayalekshmi and Rajan, 2007). As there was no dedicated cancer centre in this rural area, we had to pursue an active registration method by visiting all health-care facilities of the taluk and outside where cancer patients are seen (Jayalekshmy *et al*, 2008).

Death reports were obtained from the death registers kept in the vital statistics division of each panchayat. House visits of the deceased, to supplement information on cause of death, were started in 1997. The proportion of DCO cases in Karunagappaly cancer registry was 14% during 1990–1994 (Nair *et al*, 1997), 10% during 1993–1997 (Nair *et al*, 2002) and 4% during 1998–2002 (Jayalekshmi and Rajan, 2007). The ratio of incidence to mortality (M/I percent) for all cancer among women was 39% during the period between 2002–2003 (Jayalekshmi *et al*, 2005), similar to those in other major cancer registries in India (Nandakumar *et al*, 2005).

The extent of migration among cohort members was assessed by conducting a door-to-door survey of all the households in the six panchayats (Chavara, Neendakara, Panmana, Alappad, Oachira and Thevalakkara) and in the remaining six panchayats in 2001 and 2003, respectively. The survey findings were linked to incident cases through name, address, age, house number and so on; it showed that migration was negligible.

### Statistical analysis

Statistical analysis of tobacco chewing in relation to sociodemographic factors were conducted using logistic analysis, adjusting for age at interview. For the association with age, univariate logistic analysis was used.

Analyses of sociodemographic factors and tobacco chewing were based on the data in cross-classifications by attained age (5-year category), and other covariates. Relative risk (RR) and 95% confidence intervals (95% CI) were obtained from Poisson regression analysis of grouped survival data (Breslow and Day, 1987), using the DATAB and AMFIT procedures of Epicure programme (Preston *et al*, 1993). In the analysis of risk associated with tobacco chewing, which has the three categories (never, former and current), the following model was used to estimate the RRs of former tobacco chewers (represented by S_2_) and current-chewers (represented by S_3_): H_0_ (attained age, income) exp (*β*_2_S_2_+*β*_3_S_3_), where H_0_ represents the baseline, or background oral cancer incidence (among never smokers) for cross-classified strata by attained age and sociodemographic variables. Attained age at the time of the midpoint of 1-year interval during the observational period (1990–2005) was calculated for each cohort members by the DATAB procedure of EPICURE programme. Heterogeneity test was based on a global *P*-value for a set of indicator variables. Trend test for, for example, duration of tobacco chewing was conducted by assigning the mean duration of tobacco chewing to its each category.

## RESULTS

Among the 79 593 eligible women aged 30–84 years, 102 female cases of oral cancer (ICD9: 140, 141, 143–145) were identified by the end of 2005. After restricting the examination to women who do not smoke beedis or cigarettes and do not drink alcohol, there were 78 140 women and 92 oral cancer cases. Table 1 shows the distribution of tobacco chewers according to sociodemographic factors. All the factors examined were strongly related to tobacco chewing. Table 2 presents sociodemographic features of study subjects and the RRs for those factors obtained by the analysis stratified on attained age. The lowest family income group had a higher risk than higher income groups (*P*<0.001, the lowest income *vs* other groups).

Tables 3 and 4 summarise the results of risk analysis with respect to tobacco chewing. The analyses were stratified on attained age and family income. Tobacco chewing increased oral cavity cancer risk by 5.5-fold. Former tobacco chewers had an RR even larger than current tobacco chewers. The duration of tobacco chewing was related to incidence (*P*<0.001), particularly in the first 20 years. Among those who had chewed tobacco for 20 years or longer by the time of baseline study, no further risk increase was observed.

Table 4 summarises the results examining the effects of the daily frequency of tobacco chewing and age starting tobacco-chewing on incidence. In those analyses, those who stopped chewing tobacco by the time of interview were excluded. Oral cancer incidence was strongly related to daily frequency of tobacco chewing (*P*<0.001) and was increased 9.2-fold among women chewing tobacco 10 times or more a day. The effect of age starting tobacco chewing did not evidently modify risk. Oral cavity cancers were grouped into cancers of the tongue (ICD9: 141) and gum and mouth (ICD9: 143–145), there were only four cases in the other location, which were cancer of the lip. As shown in Table 5, tobacco chewing was significantly associated with cancers of the mouth (*P*<0.001) and the tongue (*P*<0.001).

## DISCUSSION

This study showed that daily frequency of tobacco chewing was strongly related to oral cancer incidence among women, and the risk among women chewing tobacco 10 times or more a day was 9.2-fold higher than that of non-tobacco chewers. Moreover, it increased with duration of chewing during the first 20 years.

Former tobacco chewers had an RR even larger than current tobacco chewers, as also found by a case–control study in Trivandrum, India (Muwonge *et al*, 2008). Although former chewers may include those who stopped because of precancerous lesions, the increase of risk among those who stopped 10 or more years before the interview is difficult to explain in this way.

Socioeconomic status is suspected to be related to oral cancer risk, but the results from studies have been mixed. A review concluded that most incidence studies did not show a clear association, whereas oral cancer mortality was elevated in lower SES sections of various populations (Faggiano *et al*, 1997). Recently, a case–control study in Kerala, India, showed that lower levels of education and income were related to relatively high prevalence of oral premalignant lesions (Hashibe *et al*, 2003). However, inconsistent results on SES are not unexpected, as this is most likely a surrogate marker of oral cancer, and the factors related to SES may differ from society to society. In this study, oral cancer risk among women was related to very low family income but not to education levels.

In India and Pakistan, almost 100 million people use smokeless tobacco (Reddy and Gupta, 2004), and in many ways (IARC, 2007). In most Asian countries, the widely used method is to chew ‘pan’ – a bolus made of betel leaf, areca nut or slaked lime smeared on betel leaf and tobacco. IARC has classified areca nut as a human carcinogen (group 1) (IARC, 2004). In the study area, tobacco chewing was almost always associated with chewing pan, and only a small number chewed tobacco alone, so that it was difficult to determine which was more harmful, the use of pan alone or pan together with tobacco.

A limitation of our study is the fact that the lifestyle of cohort members, may have changed during follow-up and no attempt was made to re-interview subjects. Some never-chewers at baseline may have started tobacco chewing during our follow-up period, first as some who chewed tobacco at interview may have stopped the habit during the follow-up. Our RRs for tobacco chewing may therefore be underestimated. In addition, duration of tobacco chewing and years after cessation of chewing is probably underestimated, as we used the periods until the time of interview.

This study, the first cohort study of the question among women, showed that frequent tobacco chewing strongly increases oral cancer incidence (*P*<0.001).

## Figures and Tables

**Table 1:** Tobacco chewing and sociodemographic factors

	**Tobacco chewing**				
	**Yes** a	**No** a	**Odds ratio** b	**95% CI**	
Total	18 612 (100%)	59 221 (100%)			*P*<0.001
					
*Age at interview (years)*					
30−	1889 (7%)	25 964 (93%)	1	Reference	
40−	4108 (21%)	15 377 (79%)	3.67	3.47–3.89	
50−	4757 (35%)	8734 (65%)	7.49	7.06–7.94	
60−	5060 (44%)	6414 (56%)	10.84	10.22–11.51	
70−	2411 (50%)	2411 (50%)	13.74	12.77–14.79	
80−	387 (55%)	321 (45%)	16.57	14.19–19.35	
					
*Religion*					*P*<0.001
Hindu	13 960 (25%)	41 969 (75%)	1	Reference	
Moslem	3953 (26%)	11 047 (74%)	1.18	1.13–1.23	
Christian	699 (10%)	6205 (90%)	0.31	0.28–0.33	
					
*Family income (Rs.)* a					*P*<0.001
<500	1943 (34%)	3797 (66%)	1	Reference	
501–1200	6407 (28%)	16 422 (72%)	0.79	0.74–0.84	
1201–2500	6766 (24%)	21 920 (76%)	0.60	0.56–0.63	
2501–3500	2405 (18%)	10 703 (82%)	0.40	0.37–0.42	
3500+	1091 (15%)	6379 (85%)	0.27	0.25–0.29	
					
*Education*					*P*<0.001
Illiterate	6144 (47%)	6917 (53%)	1	Reference	
Primary school	7272 (33%)	14 803 (67%)	0.67	0.64–0.70	
Middle school	3750 (20%)	14 983 (80%)	0.45	0.42–0.47	
High school	1257 (7%)	18 094 (94%)	0.22	0.21–0.24	
College	70 (2%)	4081 (98%)	0.12	0.11–0.14	
Unknown	119 (26%)	343 (74%)	0.45	0.37–0.54	
					
*Occupation*					*P*<0.001
Fishermen and farmers	1180 (53%)	1060 (47%)	1	Reference	
Unemployed	548 (13%)	3522 (87%)	0.29	0.27–0.32	
House wives/students	7523 (18%)	34 023 (82%)	0.40	0.37–0.43	
Skilled workers	9349 (32%)	20 297 (68%)	0.67	0.62–0.72	
Others	12 (4%)	319 (96%)	0.18	0.14–0.22	

a Those who chew tobacco currently or in the past. Those whose tobacco chewing status was unknown were excluded from analysis.

b Odds ratio and 95% CI (confidence interval) were obtained by logistic analysis adjusting for age at interview (5-year category). In the analysis of association with age, univariate analysis logistic analysis was conducted.

**Table 2:** Sociodemographic features of study subjects (women only)

	**Subjects (%)**	**Person-years**	**Cases** a	**RR**	**95% CI**	
Total	78 140 (100%)	921 051	92			
						
*Religion*						*P*>0.5
Hindu	56 147 (72%)	665 846	67	1	Reference	
Moslem	15072 (19)	176 024	18	1.1	0.7–1.9	
Christian	6921 (9)	79 181	7	0.9	0.4–1.9	
						
*Family income (Rs.)* a						*P*=0.401
<500	5768 (7)	71 639	13	1	Reference	
501–1200	22 939 (29)	275 136	25	0.5	0.3–1.0	
1201–2500	28 806 (37)	334 910	30	0.5	0.3–1.0	
2501–3500	13 144 (17)	150761	16	0.6	0.3–1.2	
3500+	7483 (10)	88 605	8	0.5	0.2–1.2	
						
*Education*						*P*>0.5
Illiterate	13 105 (17)	147 362	20	1	Reference	
Primary school	22 187 (28)	259 572	35	1.2	0.7–2.1	
Middle school	18 810 (24)	225 008	22	1.2	0.6–2.2	
High school	19 420 (25)	234 263	11	0.9	0.4–2.0	
College	4155 (5)	49 570	4	2.0	0.6–5.9	
Unknown	463 (1)	5276	0			
						
*Occupation*						*P*>0.5
Fishermen and farmers	2252 (3)	24 710	3	1	Reference	
Unemployed	4079 (5)	47 914	3	0.7	0.1–3.4	
House wives/students	41698 (53)	491 971	39	1.0	0.3–3.2	
Skilled workers	29 780 (38)	352 557	46	1.3	0.4–4.1	
Others	331 (0.4)	3899	1	3.3	0.3–32.3	

Relative risk (RR) and 95% CI (confidence interval) were obtained from the following model: H=H_s_ exp(*B*_*i*_*X*_*i*_), where background hazard, H_s_, was stratified by attained age (5-year category), and *X*_*i*_ are categorical variables for one of sociodemographic factors.

a Oral cancer cases.

**Table 3:** Tobacco chewing and oral cancer among women

**Tobacco chewing**	**Oral cancer case** a	**Person-years**	**RR**	**95% CI**	
*Chewing habit*					*P*<0.001
Never	25	706 872	1	Reference	
Former	14	26 804	9.2	4.6–18.1	
Current	53	183 749	5.5	3.3–9.0	
Unknown	0	3629			
					
*Duration*					*P* for trend^a^ <0.001
Never	25	706 872	1	Reference	
1–9	9	63 998	3.1	1.5–6.8	
10–19	17	38 927	8.9	4.8–16.8	
20–29	18	41 867	7.8	4.2–14.5	
30–39	14	31 439	7.1	3.6–14.1	
40+	7	31 203	3.2	1.3–7.8	
Unknown	2	6747	6.5	1.5–27.4	
					
*Years since stop tobacco chewing*					
Current smokers	53	183 849	1	Reference	
1–9	7	13 817	1.7	0.8–3.7	
10+	4	4819	2.6	0.9–7.2	
Never	25	706 872	0.2	0.1–0.3	
Unknown	3	11 796	0.8	0.2–3.3	

Relative risk (RR) and 95% confidence interval (CI) were obtained from the following model: H=H_s_ exp(*B*_*i*_*X*_*i*_), where background hazard, H_s_, was stratified by attained age (5-year category) and family income; and *X*_*i*_ are categorical variables for tobacco chewing.

a The category of ‘unknown’ was excluded when calculating *P* for trend.

**Table 4:** Tobacco chewing and oral cancer among women—former tobacco chewers are excluded from analysis

**Times**	**Oral cancer** **cases**	**Person-years**	**RR**	**95% CI**	
*Daily frequency*					
Never	25	706 872	1	Reference	
1–4	16	95 614	3.3	1.7–6.4	
5–9	25	62 143	7.8	4.4–13.9	
10+	12	25 063	9.2	4.5–18.7	
Unknown	0	4558			*P* for trend^a^ <0.001
					
*Starting age (years)*					
<20	4	21 989	3.8	1.9–7.5	
20−	15	46 775	7.8	4.2–14.4	
30−	18	49 953	6.4	3.3– 12.4	
40+	14	60 799	3.5	1.2–10.1	
Never	25	706 872	1	Reference	
Unknown	2	7862	5.7	1.3–24.3	
					*P* for trend^b^ >0.5

Relative risk (RR) and 95% confidence interval (CI) were obtained from the following model: H=H_s_ exp(*B*_*i*_*X*_*i*_), where background hazard, H_s_, was stratified by attained age (5-year category) and family income; and *X*_*i*_ are categorical variables for tobacco chewing.

a The category of ‘unknown’ was excluded when calculating *P* for trend.

b The categories of never-tobacco chewers and unknown were excluded when calculating *P*-value.

**Table 5:** Tobacco chewing and location-specific oral cancer incidence

**Site of cancer**	**Tobacco chewing**	**Oral cancer cases**	**RR**	**95% CI**	
Tongue (ICD9: 141)					*P*<0.001
	Never	13	1	Reference	
	Former	5	6.7	2.3–19.4	
	Current	20	3.9	1.9–8.0	
	Unknown	0			
Gum and mouth (ICD9: 143–145)					*P*<0.001
	Never	9	1	Reference	
	Former	9	16.7	6.3–44.0	
	Current	32	10.0	4.6–21.8	
	Unknown	0			

Relative risk (RR) and 95% confidence interval (CI) were obtained from the following model: H=H_s_ exp(B_i_X_i_), where background hazard, H_s_, was stratified by attained age (5-year category) and family income; and *X*_*i*_ are categorical variables for tobacco chewing.
